# A Paradigm Shift in Understanding the Pathological Basis of Autism Spectrum Disorder: From the Womb to the Tomb

**DOI:** 10.3390/jpm12101622

**Published:** 2022-10-01

**Authors:** Yoshihiro Noda

**Affiliations:** Department of Neuropsychiatry, Keio University School of Medicine, Tokyo 160-8582, Japan; yoshi-tms@keio.jp; Tel.: +81-3-3353-1211

Autism Spectrum Disorder (ASD) is currently diagnosed based on clinical assessment of behavioral characteristics [1]. The complexity of endophenotypes is increasing in the research on the pathophysiology of ASD, while the clinical aspect of ASD is a systemic disorder with multifaceted health needs. The diverse phenotypes of ASD and the dramatic increase in prevalence over the past few decades [2] call for a paradigm shift toward comprehensive organization of pathophysiology and multidimensional understanding of etiology of this disorder. In this editorial, I would like to outline a thought-provoking review of the recent publication by Panisi et al. entitled “Autism Spectrum Disorder from the Womb to Adulthood: Suggestions for a Paradigm Shift” [3].

The “first 1000 days” is a critical period for human neurodevelopment, and it is no exaggeration to say that the interplay and vicious cycle of immune activation, gut dysbiosis, and mitochondrial dysfunction/oxidative stress greatly affect neurodevelopment during pregnancy, which together form the early neural basis of ASD. Therefore, the most effective intervention for ASD would be primary prevention at the time of conception and early control of key effector molecular pathways. This review provides critical insights into effective prevention strategies and individualized, dynamic treatment principles for ASD from the womb to adulthood. The neurodevelopment of the embryonic/fetal brain is greatly influenced by a large number of interacting environmental factors called “exposomes”. In the womb and after birth, environmental information ultimately converges on three major interacting/overlapping pathways: (1) dysbiosis, (2) mitochondrial dysfunction/oxidative stress, and (3) immune activation including maternal immune activation (MIA) during pregnancy. Eventually, these three effector pathways directly influence the epigenetic machinery as a triumvirate of etiologies. Maternal transmission of microbiota and mitochondria [4] illustrates the importance of women’s health programs. Prenatal factors, particularly those associated with ASD, impact on development more than any other factors, and their effects are not exclusively limited to the brain. Among environmental factors, diet is also a fundamental tool in the prevention and care of ASD; patients with ASD are at increased risk for nutritional imbalances, which are, at least partially, associated with metabolic syndrome and oxidative stress [5]. In fact, in addition to energy intake, diet influences microbiota [6], immune function [7], and lipidic cell membrane profiles [8]. Individualized dietary plans may play a practical role in prevention strategies and care of ASD. In addition, appropriate diagnostic and monitoring tools are needed to properly diagnose and comprehensively understand the complexity of the condition regarding ASD.

Epigenetics, in an adaptive and predictive context, adjusts gene expression based on changes in the cellular environment to maintain homeostasis [9]. Epigenetic markers exhibit relatively high levels of neuroplasticity during the period of cellular differentiation, including neurogenesis [10], with the embryonic/fetal period and the first two years of life being the time of maximum neuroplasticity. Indeed, numerous epigenetic markers involved in epigenetic dysregulation are differentially expressed in ASD. Hence, conceptualization of a model that integrates genetics, environment, and epigenetics is required to comprehensively understand the epidemiology and clinical manifestations of ASD. The plausibility of the epigenetic paradigm for ASD is derived from the idea that the time span is too short for genetic changes to explain the drastic increase in its prevalence, and that it would be due to changes in genetic programming induced by environmental stressors that are exposed during critical periods of neurodevelopment [11]. In particular, during pregnancy, the external environment of the fetus is shaped through epigenetic molecular adaptations of the placenta. As far as neurodevelopment is concerned, the “first 1000 days” is the period when neuroplasticity is most active for neuronal proliferation, differentiation, migration, synaptogenesis, and synaptic pruning, shaping the neurodevelopmental framework for the entire lifetime [12]. In addition, a growing body of evidence suggests a close link between immune function and neurodevelopmental disorders [13]. Immune responses affect neuronal migration, synaptogenesis, white matter organization, and remodeling, some of the key steps in neural network development [14]. In fact, when the maternal body suffers from an infectious or autoimmune disease, the maternal immune response (i.e., MIA) can have a direct impact on the brain development of the fetus during pregnancy [15,16]. The impact of the current SARS-CoV-2 pandemic should be closely monitored because maternal systemic cytokine storms (mainly IL-6 and IL-17) and intrauterine inflammation may disrupt epigenetic regulatory mechanisms in the fetus [17]. In particular, MIA in late pregnancy may cause methylation modifications of key genes related to the development and function of GABAergic neurons in the fetal brain gene expression via elevated maternal cytokines and chemokines [18], which may form the pathogenetic basis of ASD. Moreover, in addition to their activity as immune mediators, IL-6 and IL-17 and others in the maternal body are involved in the migration of neuronal precursors, neuronal maintenance, synaptic pruning, and neuroplasticity [19].

On the other hand, the maternal microbiota can indirectly affect the fetus through maternal factors such as maternal immune response and microbial metabolites that pass through the placenta [20]. It can further involve diet, stress, and other factors that affect the maternal microbiota [21]. Specifically, neuroinflammation in ASD has shown an important link to the gut–brain axis (i.e., a bidirectional neuro-humoral communication system) organized by the microbiota [22]. The human gastrointestinal tract is inhabited by more than 500 species of bacteria, forming a huge amount of diversity [23]. Dysbiosis (i.e., state of disproportionate microbial communities) during the developmental period can affect the initial stages of immune system formation, which in turn shapes the wide range of neurobiological/pathological bases [24], including neurodevelopmental and psychiatric disorders, leading to subsequent adverse mental health outcomes [25]. In fact, it is known that the maternal gut microbiota during pregnancy has a strong influence on the microbiota of the infant, and the microbiota may be altered in the ASD group compared with the control group. In addition, the ASD group exhibits “leaky gut” with increased intestinal bacterial permeability compared with the control group, and the intestinal microbiota is also prone to change due to reduced intestinal barrier function, leading to a greater susceptibility to dysbiosis. This may account for the fact that approximately 20% of adult patients with ASD complain of some gastrointestinal disturbance, which has a serious impact on their well-being.

There is also accumulating evidence that mitochondrial dysfunction is closely implicated in neurodevelopmental disorders [26]. In particular, mitochondrial dysfunction and oxidative stress are involved in two major and interrelated metabolic abnormalities associated with ASD. This is because oxidative stress causes mitochondrial malfunction, and dysfunctional mitochondria produce reactive oxygen species (ROS) [27]. This intestinal motility disorder due to mitochondrial dysfunction would also explain some of the GI symptoms observed in ASD [28,29]. The balance of microbial metabolites significantly impacts on mitochondrial function, which in turn affects gastrointestinal activity. In ASD, there exists a vicious cycle among dysbiosis, immune response, and mitochondrial dysfunction/oxidative stress that initiates in the embryo/fetal stage and can affect neurodevelopment and even cause progressive deterioration of neurological function.

A dynamic and individualized approach is needed for patients with ASD. To this end, it is necessary to develop appropriate diagnostic tools that can identify the biological complexity of the condition. For example, metabolomics can describe individual molecular phenotypes and monitor their temporal changes. The molecular phenotype closely reflects the outcome of interactions among genomics, transcriptomics, proteomics, environmental factors, and the gut microbiota, and can be related to the type and extent of behavioral/cognitive impairment and the functional neuroimage [30]. Thus, a metabolomics approach holds promise in the diagnosis and follow-up of ASD and may help to understand earlier the pathophysiology of individuals with ASD who have unique medical needs. ASD is also associated with abnormal neural connectivity [31], and such brain developmental abnormalities may already be detectable at birth. Nowadays, the dynamic structure of neural networks, an indicator of neural activity, can be evaluated by time series analysis and network analysis on EEG data [32]. In other words, EEG measurements could help explain the unique symptom patterns associated with ASD characteristics. Furthermore, the application of advanced machine learning to EEG data may enable early diagnosis of ASD [33]. Such a tool could also monitor changes in neural activity with EEG measurements, find hidden etiologies associated with clinical and experimental biomarkers, and allow monitoring of the effects of therapeutic interventions.

Finally, the neural basis of ASD involves a complex interplay of genomic, epigenomic, environmental factors, and neuro-immune/endocrine interactions in a nonlinear rather than linear fashion. In fact, the epidemiological scenario has changed dramatically in recent decades, and traditional methods can no longer adequately assess the intrinsic complexity of the phenomenon, including ASD pathology. Thus, developing evaluation methods commensurate with the complexity of the phenomenon is the key to achieving personalized medicine. Specifically, we need to find an etiological mechanism that can explain epidemiology and clinical findings in a coherent manner. Toward this end, a dynamic and systematic approach, beginning with preconception women’s health care, appears to be the most promising and effective strategy for addressing this major public health problem, both in terms of current needs and future prospects.
